# The Cost of Breast Cancer: Economic and Social Perspective

**DOI:** 10.3390/cancers17183012

**Published:** 2025-09-15

**Authors:** Izabela Gąska, Aleksandra Czerw, Monika Pajewska, Olga Partyka, Andrzej Deptała, Anna Badowska-Kozakiewicz, Michał Budzik, Katarzyna Sygit, Paulina Wojtyła-Buciora, Jarosław Drobnik, Piotr Pobrotyn, Dorota Waśko-Czopnik, Julia Pobrotyn, Ewa Bandurska, Weronika Ciećko, Elżbieta Grochans, Anna M. Cybulska, Daria Schneider-Matyka, Kamila Rachubińska, Petre Iltchev, Tomasz Czapla, Remigiusz Kozlowski

**Affiliations:** 1 Medical Institute, Jan Grodek State University in Sanok, 38-500 Sanok, Poland; 2 Department of Health Economics and Medical Law, Medical University of Warsaw, 02-091 Warsaw, Poland; 3 Department of Economic and System Analyses, National Institute of Public Health NIH-National Research Institute, 00-791 Warsaw, Poland; 4 Department of Oncology Propaedeutics, Medical University of Warsaw, 01-445 Warsaw, Poland; 5 Faculty of Medicine and Health Sciences, University of Kalisz, 62-800 Kalisz, Poland; 6 Department of Family Medicine, Faculty of Medicine, Wroclaw Medical University, 51-141 Wroclaw, Poland; 7 Pulsantis Specialist and Rehabilitation Clinic Ltd., 53-238 Wroclaw, Poland; 8 Department of Gastroenterology, Hepatology with Inflammatory Bowel Disease Subunit, Provincial Specialist Hospital J. Gromkowskiego, 51-149 Wroclaw, Poland; dczopnik@gmail.com; 9 Department of Non-Surgical Clinical Sciences, Faculty of Medicine, Wrocław University of Science and Technology, 50-370 Wroclaw, Poland; 10 Faculty of Medicine, Wroclaw Medical University, 50-345 Wroclaw, Poland; 11 Center for Competence Development, Integrated Care and e-Health, Medical University of Gdansk, 80-204 Gdansk, Poland; 12 Department of Nursing, Faculty of Health Sciences, Pomeranian Medical University in Szczecin, 71-210 Szczecin, Poland; 13 Department of Management and Logistics in Healthcare, Medical University of Lodz, 90-136 Lodz, Poland; 14 Department of Management, Faculty of Management, University of Lodz, 90-237 Lodz, Poland

**Keywords:** breast cancer, BC, costs, social burden

## Abstract

**Background**: Breast cancer imposes a substantial global health and economic burden, with costs varying across disease stages, treatment modalities, and healthcare systems. Understanding these costs is crucial for informing policy and guiding efficient allocation of healthcare resources. **Objectives**: This systematic review aimed to summarize available evidence on the economic burden of breast cancer, including direct and indirect costs, and to evaluate the cost-effectiveness of treatment strategies reported in recent studies. Methods. Following PRISMA 2020 guidelines, a systematic search was conducted in MEDLINE and Academic Search Ultimate (January 2024–May 2025). Studies were included if they evaluated costs or cost-effectiveness in breast cancer patients. Screening and data extraction were performed independently by two reviewers. Data were synthesized narratively and summarized in evidence tables. **Results**: Twenty-seven studies were included. Reported costs varied widely by country, stage, and healthcare setting. In Dubai, inpatient costs reached USD 16,956 and medications USD 10,251, accounting for 84% of the disease burden. In Canada, annual excess costs per case averaged USD 27,485, with expenditures highest during the first year after diagnosis. Cost-effectiveness analyses indicated variable outcomes: toripalimab plus chemotherapy yielded an ICER of USD 16,133/QALY, suggesting potential cost-effectiveness, whereas some regimens, such as for metastatic triple-negative breast cancer, exceeded USD 500,000/QALY and were not cost-effective. Indirect costs, including productivity loss, were less frequently studied but contributed significantly to the overall burden. **Conclusions**: Breast cancer generates considerable direct and indirect costs, with marked heterogeneity across settings and stages. Standardized economic evaluations and broader inclusion of indirect costs are needed to better inform policy and resource planning.

## Simple Summary

Breast cancer is the most common cancer among women. According to estimations, the number of cases will increase in the future. Therefore, the costs associated with the disease will also increase. We conducted a literature review of the state of knowledge about the costs of treatment and the economic burden of breast cancer. We focused on the estimates of direct costs, i.e., treatment of breast cancer, adjuvant and neoadjuvant treatment, supportive care and indirect costs. A few specific combinations of drugs were cost-effective only under the condition of decreasing the unit prices of a medication. Further summarizing reviews and developing a methodology for standardized comparisons are necessary.

## 1. Introduction

Breast cancer is the most common malignant cancer among women, following lung cancer in the general population. In terms of mortality, breast cancer ranks fourth. According to information provided by the World Health Organization, the global incidence in 2022 was 1,871,979 cases. The age-standardized incidence rate was 46.8/100,000 [1]. The highest incidence rate was recorded in France at 105.4/100,000. In 2022, 666,103 mortal cases were recorded worldwide. The age-standardized mortality rate was 12.7/100,000. The probability of five-year survival is higher than 90% in high-income countries, but it is much lower in India (66%) and even lower in South Africa (40%). The lowest probability is observed in Iran, i.e., 41.6%. According to the predictions provided by International Agency for Research on Cancer [2], the estimated number of new cases will substantially increase in the coming years, particularly in Africa, Latin America and Oceania. In Africa, the number of cases is estimated to grow from 198,553 in 2022 to 459,152 in 2050. In Latin America, the number of cases is estimated to grow from 220,124 in 2022 to 350,451 in 2050. In Oceania, the number of cases is estimated to grow from 28,507 in 2022 to 46,135 in 2050. This increase translates into an increased risk of 131.2% for Africa, 59.2% for Latin America and 61.8% for Oceania, respectively, when compared to 2022 as a case base.

The main risk factors for breast cancer include advancing age, starting menstruation before age 12, beginning menopause after age 55, having dense breasts, a personal history of breast cancer or non-cancerous breast conditions, a family history of breast or ovarian cancer, and prior radiation therapy treatment [3]. Diagnostic and screening tests include mammography, ultrasound, and breast magnetic resonance imaging (MRI) and biopsy [4]. The treatment methods of breast cancer include mastectomy, hormone therapy, targeted therapy, chemotherapy, immunotherapy and radiation therapy [5,6,7].

Breast cancer is a chronic disease and it requires long-term treatment, which is expensive and usually limits the social and professional life of patients. The diagnosis affects entire families, causing emotional distress and requiring family members to take on caregiving responsibilities. This emotional toll extends to patients’ social lives, where they report withdrawing from social activities due to physical weakness, treatment side effects, and emotional distress, further diminishing their quality of life. Moreover, breast cancer imposes substantial financial strain on patients and families, with survivors often experiencing worsened financial status during and after treatment despite assistance; one study found financial distress scores remained significantly elevated post-treatment [8]. Two types of costs should be taken into consideration, direct and indirect costs.

The first category of costs primarily includes the healthcare system’s expenses for medical services, as well as patients’ out-of-pocket costs for treatment, such as expenses for medications and medical supplies. The second category covers the costs of lost productivity due to sickness absence or presenteeism [9,10]. According to projections from the Global Cancer Observatory, the incidence of breast cancer is expected to rise, leading to an increase in the number of patients, higher treatment costs, and a growing loss of productivity from sickness-related absences. This article aims to present the latest available knowledge on both the direct and indirect costs of breast cancer, highlighting the increasing financial strain on healthcare systems.

Since breast cancer is a serious disease regarding prevalence, costs and prognosis and new research projects on the matter are published every year, we found it important to summarize their findings. Therefore, we aimed to provide answers to the following research question: what are the current direct and indirect costs associated with breast cancer according to latest research and what types of treatment were found to be cost-effective? We focused on the estimates of direct costs, i.e., treatment of breast cancer, adjuvant and neoadjuvant treatment, supportive care and indirect costs. Also, we aimed to provide a response to the following research question: what types of costs are mostly focused on in the latest research and what type of gap exists in the published literature.

## 2. Materials and Methods

A literature review was conducted to summarize the state of knowledge about the costs of treatment and the economic burden of breast cancer within MEDLINE and Academic Search Ultimate databases. We applied the following query: (breast cancer) AND (cost OR costs OR economic analysis OR economic evaluation OR economic loss OR expenditure OR spend OR expense OR burden OR productivity OR costs OR cost analysis). We limited the scope to papers published in 2024 and 2025. We also applied additional filters limiting the papers acquired to the ones focused on humans and providing the answers to clinical questions focusing on costs and economics. The study was registered in the PROSPERO system under the number CRD420251131809. The criteria for inclusion of publications according to the PICOS scheme are presented in Table 1.

We included papers that were based on Markov model simulations, partitioned survival models, medical records, surveys or systematic reviews.

Markov models in the field of health economic evaluation provide means to estimate the probability of transition to other health states, costs and health benefits using the current data as a basis and taking the perspective focused on the future of patients, their quality of life and associated costs [11]. Also, Markov models can take the probability of transition from one health state to another into account as well as the fact that costs can vary over time.

The partitioned survival models are frequently used within oncology modelling [12]. Within this framework, the population is partitioned into different health states. The process of moving between a mutually exclusive set of health states is examined. The difference between partitioned survival models and Markov models is that the former estimates the proportions in each state based on parametric survival equations. The results acquired in clinical trials are substituted into these equations. The main advantage is the ability to estimate long-term effects from studies limited in duration.

## 3. Results

**Figure 1:**
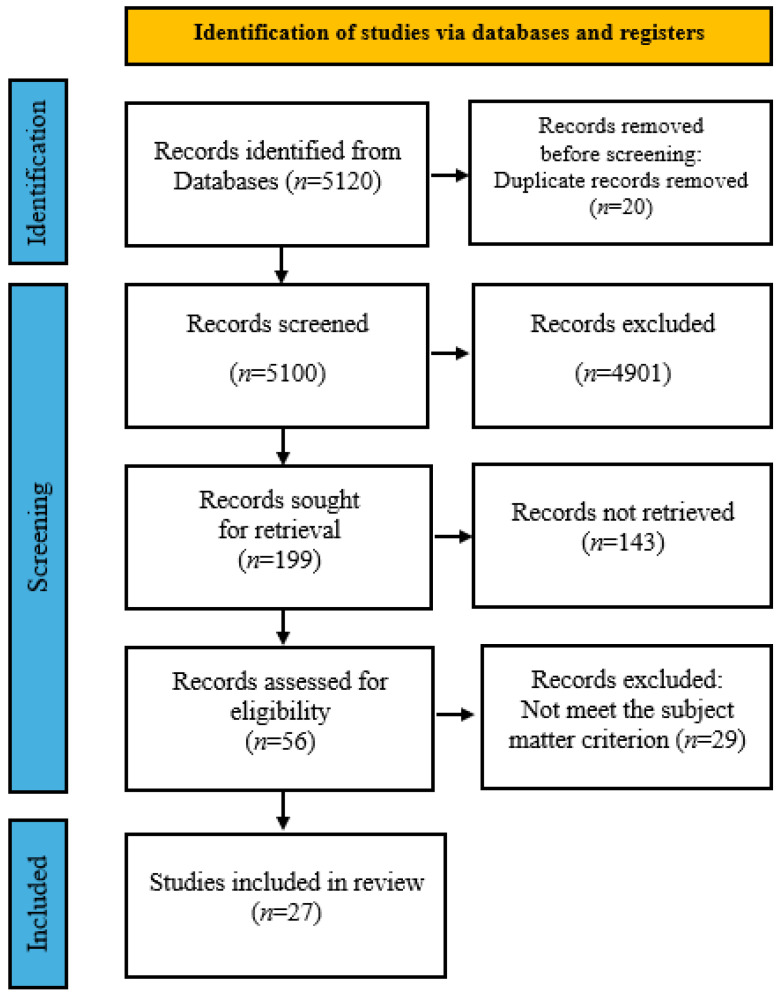
Simplified PRISMA scheme for inclusion of publications for further review.

The PRISMA schema is depicted in Figure 1.

The search performed within the records of databases led to 199 papers. After title and abstract verification of relevancy to the subject, we limited the number of articles to 56. Next, full-text verification was performed and it ended up with 27 papers included in the final analysis. For this, two authors evaluated the abstracts of the publications, qualifying them for further analysis. Potentially eligible publications were then independently reviewed by another two authors. All doubts regarding further inclusion of the publications in the review were resolved by consensus of the entire team of authors.

We excluded the studies devoted to breast cancer screening, studies focused on other types of cancer and studies focused on survival rates.

Table 2 presents a summary of the publications included in the review with a description of the most important features. The studies were published between 2024 and 2025, so only the latest research projects were included. Five papers originate from the USA, four from China and two from Iran. Also, they are papers that originate from Finland, Taiwan, United Arab Emirates, Spain, Sweden, Sri Lanka, Canada and India, with one paper for each. Table 2 provides information regarding the unit of measure used, type of methodology, type of costs and group of patients each study was focused on. The units of measures are currency, ICER (Incremental Cost-Effectiveness Ratio), which is the difference in costs divided by the difference in health outcomes, or QALY (Quality Adjusted Life Years), which combines the value of length of life and quality of life into a single index number.

The review of the results acquired in the studies included is divided into sections for direct costs, i.e., treatment of breast cancer, adjuvant and neoadjuvant treatment, supportive care and indirect costs. It gives answers to our first research question as it provides the results regarding costs of breast cancer and cost-effectiveness analysis. In the following sections, we provide estimates in the currency used by the authors of the original papers. Also, if the authors used currency other than USD, we converted it into USD using the annual mean of operational rates of exchange. These rates are provided by the United Nations Treasury [13].

**Table 2:** Characteristics of publications included in the review.

Author/Year	Country	Unit of Measure	Methodology	Type of Costs	Group of Patients
Yaghoubi, N. et al., 2025 [14]	Iran	ICER, QALY	Cost-effectiveness analysis	Direct costs	Patients with metastatic breast cancer
Paulissen, J. et al., 2024 [15]	Finland	ICER, QALY	Assessment of the cost of the disease	Direct costs	Patients with HER2-positive (HER2+) unresectable and/or metastatic breast cancer
Heng, J. et al., 2024 [16]	Malaysia	USD	Cost-effectiveness analysis	Direct costs	Patients with breast cancer
Khoirunnisa, S.M. et al., 2024 [17]	Indonesia	USD	Cost-effectiveness analysis	Direct costs	Patients with HER2-positive (HER2+) breast cancer
Seyedifar, M. et al., 2024 [18]	IRAN	ICER, QALY	Cost-effectiveness analysis	Direct costs	Patients with HER2-positive (HER2+) breast cancer
Jia, C. et al., 2025 [19]	China	ICER, QALY	Cost-effectiveness analysis	Direct costs	Patients with Hormone receptor HR-positive/HER2-negative (HR+/HER2−) breast cancer
Nguyen, T.T.H et al., 2025 [20]	United States	ICER, QALY	Cost-effectiveness analysis	Direct costs	Patients with HER2-positive (HER2+) breast cancer
Chang, S. et al., 2024 [21]	United States	ICER, QALY	Cost-effectiveness analysis	Direct costs	Patients with Hormone receptor HR-positive/HER2-negative (HR+/HER2−) breast cancer
Sra, M. et al., 2024 [22]	India	ICER, QALY	Cost-effectiveness analysis	Direct costs	Patients with Hormone receptor HR-positive/HER2-negative (HR+/HER2−) breast cancer
Pan, J. et al., 2024 [23]	United States, China	ICER, QALY	Assessment of the cost of the disease	Direct costs	Patients with HER2-negative (HER2−) breast cancer
Xu, C. et al., 2025 [24]	United States, China	QALY	Cost-effectiveness analysis	Direct costs	Patients with HER2-negative (HER2−) breast cancer
Wang, L. et al., 2024 [25]	China	ICER, QALY	Cost-effectiveness analysis	Direct costs	Patients with metastatic triple-negative breast cancer
Cai, H. et al., 2024 [26]	China	ICER, QALY	Cost-effectiveness analysis	Direct costs	Patients with metastatic triple-negative breast cancer
Chen, P. et al., 2025 [27]	United States	ICER, QALY	Cost-effectiveness analysis	Direct costs	Patients with metastatic triple-negative breast cancer
Tseng, T. et al., 2024 [28]	Taiwan	ICER, QALY	Cost-effectiveness analysis	Direct costs	Patients with breast cancer
Mok, C. et al., 2025 [29]	Singapore	ICER, QALY	Cost-effectiveness analysis	Direct costs	Patients with metastatic breast cancer
Kim, Y. et al., 2024 [30]	United States	USD	Assessment of the cost of the disease	Direct costs	Patients with breast cancer who underwent breast-conserving surgery
Reddy, K. et al., 2025 [31]	United States	USD	Assessment of the cost of the disease	Direct costs	Patients with breast cancer
Teli, B. et al., 2025 [32]	Iran	USD	Assessment of the cost of the disease	Direct and indirect costs	Patients with breast cancer
Irandoust, K. et al., 2025 [33]	Iran	USD	Assessment of the cost of the disease	Direct costs	Patients with breast cancer
Hamza, D. et al., 2024 [34]	United Arab Emirates	USD	Assessment of the cost of the disease	Direct costs	Patients with breast cancer
Mittmann, N. et al., 2024 [35]	Canada	USD	Assessment of the cost of the disease	Direct costs	Patients with breast cancer
Malhan, S. et al., 2024 [36]	Turkey	USD	Assessment of the cost of the disease	Direct and indirect costs	Patients with breast cancer
Gunasekara, A. et al., 2024 [37]	Sri Lanka	ICER	Cost-effectiveness analysis	Direct costs	Patients with HER2-positive (HER2+) breast cancer
Fenix-Caballero, S. et al., 2025 [38]	Spain	USD, QALY	Cost-effectiveness analysis	Direct costs	Patients with HER2-positive (HER2+) breast cancer
Crafoord, M. et al., 2025 [39]	Sweden	ICER, QALY	Cost-effectiveness analysis	Direct costs	Patients with breast cancer undergoing neoadjuvant chemotherapy
Franklin, M. et al., 2024 [40]	United States	USD	Assessment of the cost of the disease	Indirect costs	Patients with breast cancer

### 3.1. Direct Costs

#### 3.1.1. Treatment of Breast Cancer

A cost effectiveness study [14] demonstrated Trastuzumab deruxtecan to be a cost-effective alternative to trastuzumab emtansine as a second-line treatment for cases with human epidermal growth factor receptor 2 (conventionally labelled HER2) overexpression, i.e., 20% of breast cancer cases with worse overall survival outcomes. The HER2 receptor is a type of oncoprotein involved in cell proliferation protecting abnormal cancer cells from apoptosis, which is programmed cell death. Trastuzumab deruxtecan is an antibody–drug conjugate targeting HER2. It was associated with 3.59 QALYs for USD 261,315, and trastuzumab emtansine with 1.89 QALYs at USD 258,039. This results in an ICER of USD 1927 per QALY and it was below the willingness-to-pay threshold equal to USD 2413/QALY.

The treatment with Trastuzumab deruxtecan was also evaluated for the group of patients with HER2+ unresectable and/or metastatic breast cancer [15]. Total quality-adjusted life years and life years gained for Trastuzumab deruxtecan compared with ado-trastuzumab emtansine were equal to 1.93 and 2.56, respectively. Incremental costs associated with Trastuzumab deruxtecan were EUR 106,800, which can be converted into USD 115,454.26, resulting in an ICER of EUR 55,360 per QALY gained, which can be converted into USD 59,845.95 and an ICER of EUR 41,775 per life years gained, which can be converted into USD 45,160.13.

Treatment based on biosimilar intravenous trastuzumab was more economical than treatment based on subcutaneous trastuzumab in Malaysia [16]. The mean total costs were equal to USD 5624 and USD 13,693 and per patient, respectively. This study also evaluated treatment based on antibody that inhibits the growth of cancer cells by interfering with the HER2 receptor. The records of all HER2+ breast cancer patients were included in the analysis.

In Indonesia, treating HER-2 positive early breast cancer with trastuzumab and chemotherapy was compared to chemotherapy alone [17]. ICER was equal to USD 6842/QALY and to USD 5510 for a life year gained.

Trastuzumab-Emtansine was found to be close to cost-effectiveness in Iran for HER2+ breast cancer [18]. The additional cost of USD 1408 yielded a 1.59 QALY increase and it was above the threshold of USD 1085 in Iran.

In the four studies cited, above Trastuzumab or Trastuzumab deruxtecan was found to be cost-effective treatment for patients with HER2+ breast cancer. Trastuzumab-Emtansine, an alternative to Trastuzumab in addressing resistance to trastuzumab, demonstrated close cost-effectiveness.

CDK4/6 inhibitors, i.e., palbociclib, ribociclib and abemaciclib, were proven to be effective in treating HR-positive/HER2-negative (HR+/HER2−) breast cancer [19], i.e., for cases where the cancer cells have receptors for estrogen and/or progesterone (HR+) but do not have high levels of the HER2 protein (HER2−). The mechanism of action is based on inhibiting the phosphorylation of tumor-suppressor retinoblastoma protein by preventing CDK4/6 from binding to cyclin D. This effectively inhibits tumor cell proliferation, and delay or even overcome the development of endocrine resistance. The benefits were estimated to be equal to 2.10 QALYs for palbociclib plus fulvestrant, 2.55 QALYs for ribociclib plus fulvestrant, and 2.60 QALYs for abemaciclib plus fulvestrant. The costs were equal to USD 34,423, USD 41,119, and USD 48,019, respectively. Compared with palbociclib plus fulvestrant, the ICERs were USD 27,161 per QALY for abemaciclib plus fulvestrant and USD 15,073 per QALY for ribociclib plus fulvestrant. The willingness-to-pay threshold was equal to USD 37,738.

The addition of capivasertib to fulvestrant in treating HR-positive/HER2-negative (HR+/HER2−) breast cancer was associated with an additional cost of USD 410,765 yielding 1.46QAL [20]. ICER was equal to USD 280,854/QALY. This strategy was not cost-effective when compared to the willingness-to-pay threshold equal to USD 100,00/QALY. Capivasertib inhibits AKT (protein kinase B) isoforms by blocking their catalytic activity and prevents the phosphorylation of its downstream substrates, which are proteins that control vital cell processes like cell division, metabolism, and the cell cycle. This slows or stops the growth and spread of cancer cells.

A study [21] found that administering abemaciclib, which is a CDK4/6 inhibitor in the early stage of HR-positive/HER2-negative (HR+/HER2−) breast cancer, rather than waiting until patients develop metastatic disease, was a cost-effective treatment strategy. Early treatment was associated with 21.08 life-years (LYs) and 17.93 quality-adjusted life-years (QALYs) for a cost of USD 586,213, whereas delayed treatment resulted in 11.14 LYs and 9.38 QALYs for USD 157,576. The incremental cost-effectiveness ratio (ICER) of early vs. delayed treatment was USD 43,136 per LY and USD 50,104 per QALY. The willingness-to-pay threshold was equal to USD 100,000/QALY.

The use of abemaciclib and ribociclib, CDK4/6 inhibitors, with letrozole in treatment of HR-positive/HER2-negative (HR+/HER2−) breast cancer was also evaluated in India taking two scenarios into consideration [22]. A best-case scenario was the benefit of treatment that lasts a lifetime. A worst-case was where the benefits disappear after 5 years. In the best-case scenario, abemaciclib added 2.17 QALY and 4.96 LY, involving USD 27,756.65 additional costs. In the BC scenario, ribociclib added 0.98 QALY and 2.58 LY with added costs of USD 20,494.6.

Talazoparib was evaluated for treating advanced HER2− breast cancer with germline BRCA1/2 mutations from the perspective of the health sector in China and in the United States [23]. The incremental cost in China was USD 2484.48/QALY, with an incremental QALY of 1.5. In the United States, the use of Talazoparib was associated with saving costs of USD 10,223.43 and increasing QALYs by 1.5. In the case of BRCA1/2 mutations, cells rely on DNA single-strand repair instead of double-strand, which is regulated by adenosine diphosphate ribose polymerase (PARP). Talazoparib, which is a PARP inhibitor, makes damaged DNA repair ineffective and induces the death of breast cancer cells.

Olaparib, another PARP inhibitor with positive outcomes in treating HER2− early-stage cancer, was not found to be cost-effective in the United States or in China [24]. The expenditure was equal to USD 245,604.01, yielding 7.53 QALYs, and to RMB 384,274.75, which can be converted into USD 53,086.05, yielding 6.41 QALYs.

In the six studies cited above regarding patients with HER2− breast cancer, CDK4/6 inhibitors, specifically abemaciclib, and talazoparib, a PARP inhibitor, were shown to be cost-effective.

Sacituzumab govitecan was proven to be not cost-effective for patients with metastatic triple-negative breast cancer compared with chemotherapy [25]. Metastatic triple-negative breast cancer is associated with poor prognosis and survival outcomes. Sacituzumab govitecan, a newly approved medicine, is an antibody–drug conjugate targeting trophoblast cell-surface antigen 2 and has a significant killing effect on tumor-targeted property. However, its use provided an additional 0.25 QALYs at an incremental cost of USD 81,778.61 compared to chemotherapy, resulting in an incremental cost-effectiveness ratio (ICER) of USD 323,603.84 per QALY.

For the same population of patients with metastatic triple-negative breast cancer, toripalimab, an immune checkpoint inhibitor, was demonstrated to be cost-effective [26]. This inhibitor targets key molecules that regulate immune responses, prevent excessive immune activation and enhance anti-tumor immune responses. Treatment with the toripalimab regimen resulted in a gain of 0.74 QALYs and an incremental cost of USD 11,938.55 compared with a placebo plus chemotherapy, yielding an ICER of USD 16,133.18 per QALY. The willingness-to-pay threshold was defined as USD 39,855.79 per QALY.

However, in another study [27], Toripalimab combined with nab-P chemotherapy for patients with metastatic triple-negative breast cancer associated with an additional 2.68 life-years and 1.72 QALYs, with an ICER of USD 593,750 per QALY, and did not demonstrate a significant cost-effectiveness advantage over nab-paclitaxel chemotherapy without Toripalimab.

Out of the three studies regarding treatment of patients with triple-negative breast cancer, only one concluded that one type of treatment (Toripalimab plus chemotherapy) was cost-effective.

Table 3 presents a summary of the conclusions from the studies evaluating pharmacological treatment.

The use of granulocyte colony-stimulating factor for preventing febrile neutropenia in the course of the treating breast cancer with high-risk chemotherapy treatment was evaluated [28]. Primary prevention with the use of pegfilgrastim had an ICER of NT USD 269,683, which can be converted into USD 8815.78, per quality-adjusted life year (QALY) gained compared to prevention with lenograstim. The ICER for primary prevention with lenograstim versus no granulocyte colony-stimulating factor prophylaxis was NT USD 61,995, which can be converted into USD 2026.58, per QALY gained.

Robotic mastectomy was found to be associated with higher upfront costs, but also higher long-term QALY gains when compared to conventional mastectomy [29]. The ICER for RM was estimated at SGD 30,000, which can be converted into USD 22,811.38, per QALY.

The costs of reoperations in the population of patients with breast cancer who underwent breast-conserving surgery were estimated in another study [30]. Overall reoperation rates were estimated to be equal to 21.1% for the commercial service and 14.9% for Medicare. The mean healthcare costs during 1 year of follow-up from the initial breast-conserving surgery were equal to USD 95,165 for public medical care and USD 36,313 for Medicare. Reoperations were associated with 24% higher costs in both groups, corresponding to incremental costs of USD 21,607 and USD 8559, respectively.

The cost associated with delayed or forgone care of breast cancer was also estimated [31]. Patients suffering from delayed or forgone care had USD 5372 (95% CI USD 35-USD 10,709) higher per capita inpatient expenditures, especially due to higher number of hospitalizations. Lack of adequate financial resources was the most cited reason for delaying treatment.

In a retrospective cost-of-illness analysis involving medical history of 525 patients in Iran, the total economic burden of breast cancer was estimated to be equal to USD 5394,409.13, with a mean of USD 10,275.07 per patient [32]. A systematic review [33] provided an estimation of the average annual direct medical costs per patient with breast cancer that varied USD 13,954 to USD 34,772.

A cost-of-illness analysis of breast cancer patients in Dubai [34] reported inpatient costs of USD 16,956.2 and medication costs of USD 10,251.3, with breast cancer-specific expenditures accounting for 84% of the overall disease burden.

In Canada [35], breast cancer was associated with an additional annual cost of USD 27,485 per case compared with matched controls (matched by birth year, local health integrative network, income quintile, and baseline resource utilization). Costs varied by disease stage, ranging from USD 15,588 for stage I to USD 137,319 for stage IV. The highest expenditures occurred in the first 12 months following diagnosis (USD 43,408), followed by the last 12 months of life (USD 25,940). Costs were also higher when diagnosis occurred before age 70 (USD 28,415) compared with diagnoses at age 70 or older (USD 25,254).

In a study conducted in Türkiye, the mean annual cost for newly diagnosed breast cancer was estimated to be equal to USD 21,595.62 for a patient if cancer was metastatic and USD 4490.76 for a patient if cancer was non-metastatic [36].

HR-positive/HER2-negative (HR+/HER2−) breast cancer were the most frequent diagnostic categories taken into consideration. According to the studies reviewed above, some specific combinations of drugs may be cost-effective only under the condition of dropping the prices of a medication.

#### 3.1.2. Adjuvant and Neoadjuvant Treatment

The cost-utility of neoadjuvant treatment with the use of trastuzumab plus pertuzumab or lapatinib, another monoclonal antibody, compared to single therapy with trastuzumab and chemotherapy was analysed [37] from the perspective of five-year budget impacts regarding the population of patients with HER2+ breast cancer. ICER for neoadjuvant lapatinib plus trastuzumab plus chemotherapy plus adjuvant trastuzumab was cost-effective and equal to USD 2716.

Abemaciclib, an CDK4/6 inhibitor, for the adjuvant treatment of luminal HER2 was proved not to be cost effective in Spain [38]. Total costs were EUR 98,765 for abemaciclib plus hormone therapy, which can be converted into USD 109,293.62, and EUR 17,935, which can be converted into USD 19,846.92, for hormone therapy alone. The health outcome was 10.076 QALY for abemaciclib plus hormone therapy and 9.495QALY for hormone therapy.

Only two studies were focused on adjuvant or neoadjuvant treatment and both were country specific, with from Sri Lanka and the other from Spain.

#### 3.1.3. Supportive Care

Computer application management of electronic patient-reported outcomes was evaluated in terms of cost-effectiveness [39]. The use of electronic patient-reported outcomes is supposed to facilitate early detection of symptoms and timely symptom management. However, it was associated with only a small improvement in QALYs, even if the intervention cost was low. Specifically, the mean intervention cost was equal to EUR 92, which can be converted into USD 101,81. The mean cost for the intervention and all healthcare was EUR 36,882, which can be converted into USD 40,813.72 with an ICER equal to EUR 202,368 that can be converted into USD 223,940.98.

### 3.2. Indirect Costs

In a retrospective cost-of-illness analysis involving medical history of 525 patients [32] in Iran, the indirect costs of breast cancer were estimated to be equal to USD 933,232.78, which is equal to 17.3% of the total economic burden. In a systematic review focused on estimating the economic burden of breast cancer in the United States, Canada, Australia, and Western Europe [40], indirect costs were estimated to be equal to fall within the wide range from USD 1031 to USD 29,753 with a median equal to USD 2443. The productivity loss associated with the diagnosis of operable breast cancer was reported to be equal to USD 29,753 regarding human capital per person per year and equal to USD 10,114 regarding human resource friction [32]. The cost of sick leave for patients was estimated from the French National Health Insurance perspective only and it was equal to USD 11,577 per person per year.

In a study conducted in Türkiye, the total indirect cost was estimated to be equal to USD 982,867,753.58, USD 815,199,359.02 for newly diagnosed patients and USD 169,767,030.43 for patients diagnosed previously [36].

## 4. Discussion

The majority of papers were focused on direct costs of treatment. Most studies were focused on comparisons within the same-drug class, and the majority of them led to favorable conclusions. This finding is consistent with another review on the topic [41]. Only 2 out of 22 studies were focused on adjuvant and neoadjuvant treatment and also just 2 on indirect costs. This shows a literature gap for evaluating indirect costs of breast cancer, the type of costs mostly overlooked. In addition, this conclusion is the answer to the second research question of our review. The studies focused on direct costs of treatment took specific types of diagnosis into consideration. HR-positive/HER2-negative (HR+/HER2−) breast cancer were the most frequent categories. A few studies revealed that a specific combination of drugs is cost-effective only under the condition of dropping the unit prices of a medication. Nine studies were based on the results acquired with the use of Markov models and five on the results acquired with the use of partitioned survival models. Five studies were based on medical records, two on surveys and two were systematic reviews.

The comparison of different treatments regarding cost effectiveness is difficult because of complexity. Different studies use different units for assessing the costs, especially when comparing different countries, but also the cost effectiveness depends on the precise diagnostic category and on the stage of a disease. The same is true for indirect costs as the estimations yield wide confidence intervals.

The estimated numbers of new cases of breast cancer are supposed to grow in the coming years, which inevitably will lead to an increase in all types of costs. Therefore, summarizing reviews and developing a methodology for standardized comparisons are necessary.

Our review is limited. Firstly, the scope of this paper based on the systematic review methodology is limited to the papers included in the MEDLINE database only. Moreover, the evaluations of direct costs from different studies use different units depending on the currency specific to the region studied in each paper and this impedes comparisons because the relative value of currency when exchanged to another currency changes over time. Also, the accessibility of papers focused on indirect costs is limited as they are all country-specific, which is a key limitation as well.

## 5. Conclusions

Current evidence indicates that cost-of-illness studies on breast cancer mostly concentrate on direct medical expenditures, while indirect costs and broader societal consequences remain sparsely examined. This imbalance limited a full estimate of the disease’s economic burden and restricted cross-study comparability. The article therefore underscores the need for more comprehensive investigations that integrate both direct and indirect cost components and adopt standardized costing frameworks. Such methodological harmonization would improve the accuracy of economic estimates and guide more informed resource allocation. Addressing these research gaps is essential for shaping cost-effective policies and ultimately enhancing the management and system response to breast cancer.

The breast cancer incidence will increase in the future. At the same time, access to appropriate treatment may remain different depending on the level of income in a country. For example, in China, the out-of-pocket money paid by patients for health in 2022 is USD 381, USD 125 in India, USD 551 in Poland, USD 1.380 in the USA and USD 378 in Iran [42]. Furthermore, the out-of-pocket cost increases over the course of treatment [43]. The increasing costs can have a negative impact on a patient’s life leading to financial insecurity. A 2022 survey by Breastcancer.org of 1437 Americans diagnosed with breast cancer in the past 10 years found that 47% felt their breast cancer-related out-of-pocket costs were a “significant or catastrophic burden”, 37% reduced spending on basic necessities to pay for treatment and 28% used credit cards to pay for cancer care [44]. Therefore, regarding indirect costs, financial toxicity should also be examined further in future research.

## Figures and Tables

**Table 1:** Criteria for inclusion of publications according to the PICO scheme.

Population (P)	Patients diagnosed with breast cancer
Intervention (I)	Economic factors, including costs and economic impact
Comparator (C)	Any comparator or no comparator
Outcomes (O)	Direct costs of breast cancer treatment, indirect costs, and the overall economic burden of breast cancer
Studies (S)	Case studies, prospective studies, retrospective studies, systematic review, and randomized controlled trials (RCTs)
Limitations	Publications in English that assess the impact of breast cancer on quality of life, with a publication date range from January 1, 2024, to May 30, 2025
Exclusion	Non-English publications and studies not directly related to breast cancer

**Table 3:** Summary of the conclusions from the studies evaluating pharmacological treatment.

Publication Number *	Diagnosis	Stage	Mechanism of Action	Treatment	Cost-Effectiveness
[14]	(HER2)-positive metastatic breast cancer	Second line treatment	Antibody that inhibits the growth of cancer cells by interfering with the HER2 receptor	Trastuzumab deruxtecan	+
[15]	(HER2)-positive unspecified or metastatic breast cancer	Second line treatment	Antibody that inhibits the growth of cancer cells by interfering with the HER2 receptor	Trastuzumab deruxtecan	+
[16]	(HER2)-positive breast cancer	All HER2+ breast cancer patients	Antibody that inhibits the growth of cancer cells by interfering with the HER2 receptor	biosimilar intravenous Trastuzumab	+
[17]	HER2-positive early breast cancer	Early	Antibody that inhibits the growth of cancer cells by interfering with the HER2 receptor	Trastuzumab + chemotherapy	+
[18]	(HER2)-positive breast cancer	Residual	Antibody that inhibits the growth of cancer cells by interfering with the HER2 receptor	Trastuzumab-emtansine	+/−
[19]	HR+/HER2- breast cancer	Advanced or metastatic	Inhibiting the phosphorylation of tumor suppressor retinoblastoma protein by preventing CDK4/6 from binding to cyclin D	CDK4/6 inhibitors	+
[20]	HR+/HER2- breast cancer	Advanced	Inhibiting AKT (protein kinase B) isoforms	Capivasertib + Fulvestrant	−
[21]	HR+/HER2- breast cancer	Early	Inhibiting the phosphorylation of tumor suppressor retinoblastoma protein by preventing CDK4/6 from binding to cyclin D	abemaciclib	+
[22]	HR+/HER2- breast cancer	Early	Inhibiting the phosphorylation of tumor suppressor retinoblastoma protein by preventing CDK4/6 from binding to cyclin D	Abemaciclib, Ribociclib	−
[23]	Germline BRCA1/2 mutated HER2−	Advanced	Inhibiting adenosine diphosphate ribose polymerase	Talazoparib	+
[24]	HR+/HER2− breast cancer	Early	Inhibiting adenosine diphosphate ribose polymerase	Olaparib	−
[25]	Triple-negative breast cancer	3 health states	Antibodies targeting trophoblast cell-surface antigen 2	Sacituzumab govitecan	−
[26]	Triple-negative breast cancer	Second line treatment	Immune checkpoint inhibitor regulating immune responses	Toripalimab + chemotherapy	+
[27]	Triple-negative breast cancer	3 health states	Immune checkpoint inhibitor regulating immune responses	Toripalimab + chemotherapy	−

* Publication number based on Table 2.

## Data Availability

No new data were created or analyzed in this study. Data sharing is not applicable to this article.
